# Patient‐defined goals for the treatment of severe aortic stenosis: a qualitative analysis

**DOI:** 10.1111/hex.12393

**Published:** 2015-08-14

**Authors:** Megan Coylewright, Roseanne Palmer, Elizabeth S. O'Neill, John F. Robb, Terri R. Fried

**Affiliations:** ^1^ Section of CardiologyDepartment of MedicineDartmouth‐Hitchcock Medical CenterLebanonNHUSA; ^2^ The Dartmouth Institute for Health Policy and Clinical PracticeGeisel School of Medicine at DartmouthLebanonNHUSA; ^3^ Department of Internal MedicineSchool of MedicineYale UniversityNew HavenCTUSA; ^4^ Clinical Epidemiology Research CenterVeterans Affairs Connecticut Healthcare SystemWest HavenCTUSA

**Keywords:** aortic stenosis, aortic valve replacement, elderly, patient‐centred care, shared decision making, transcatheter aortic valve implantation, valve replacement, valvular heart disease

## Abstract

**Background:**

Patients with severe aortic stenosis (AS) at high risk for aortic valve replacement are a unique population with multiple treatment options, including medical therapy, surgical aortic valve replacement and transcatheter aortic valve replacement (TAVR). Traditionally, in elderly populations, goals of treatment may favour quality of life over survival. Professional guidelines recommend that clinicians engage patients in shared decision making, a process that may lead to decisions more aligned with patient‐defined goals of care. Goals of care for high‐risk patients with AS are not well defined in the literature, and patient‐reported barriers to shared decision making highlight the need for explicit encouragement from clinicians for patient involvement.

**Objective:**

The purpose of this study was to elicit and report patient‐defined goals from elderly patients facing treatment decisions for severe AS.

**Methods:**

This analysis was conducted at Dartmouth‐Hitchcock Medical Center, an academic medical institution. In a retrospective manner, we qualitatively analysed goal statements reported by high‐risk, elderly patients with severe AS evaluated for TAVR between June 2012 and August 2014.

**Results:**

Forty‐six patients provided treatment goals during consideration of TAVR and defined preferred outcomes as maintaining independence, staying alive, reducing symptoms or, most commonly, increasing their ability to do a specific activity or hobby.

**Conclusions:**

In the high‐risk patient population considering TAVR, patient‐reported goals may be obtained with a simple question delivered during the clinical encounter. Encouraging patients to define their goals may lead to a greater degree of shared decision making, as advocated in current professional guidelines.

## Introduction

Aortic stenosis (AS) is the most common valvular heart disease in the developed world, primarily affecting the elderly. Once symptomatic, mortality rates for patients with severe AS at high risk for surgery approach 30–50% at one year if valve replacement is not performed.^1^

Several treatment options are available to patients with severe AS including medical therapy, surgical aortic valve replacement (SAVR) and transcatheter aortic valve replacement (TAVR). Medicines help to alleviate symptoms initially, but will not prevent or delay disease progression; medical therapy is a palliative therapeutic option. Among patients with severe, symptomatic AS who are either inoperable or high‐risk for traditional SAVR, TAVR is a less invasive therapy shown to achieve comparable clinical outcomes, with differences in specific risks, benefits and recovery time.^2^, ^3^

Current professional guidelines call for clinicians to utilize shared decision making when two comparable, but distinctly different, treatment options exist for valvular heart disease.^4^ Shared decision making requires a discussion between the clinician and the patient about treatment options framed by the patient's preferences and values, as well as by risks and benefits.^5^ Although barriers to implementation of shared decision making are well‐defined,^6^ studies suggest that various interventions may encourage patient participation within the clinical encounter. A synthesis of the results of 46 studies examining the effectiveness of ‘patient‐targeted interventions’ suggests that such interventions may have an effect on patient participation in cancer care consultations.^7^ A systematic review of 16 articles suggests that question prompt lists may positively affect patient participation by promoting question asking; such lists may improve cognitive and ‘psychological outcomes’ like information recall and decreased anxiety over time, respectively.^8^ Strategies for preference elicitation vary among clinical encounters and disease conditions:^9^ a Cochrane review of 115 diverse decision aid studies demonstrated that decision aids reduce decisional conflict, increase patient knowledge and are associated with improved physician‐patient communication within the medical visit, suggesting that there may be multiple successful strategies to patient engagement.^10^

There remains a documented need for ‘explicit encouragement’ of the patient by the clinician to be involved in decision making, particularly outside of rigorous controlled trials.^11^ There is evidence that even the most clinically astute physicians continue to make inaccurate assumptions about patient values and preferences, particularly in a medical culture that is focused on a ‘disease‐outcome‐based paradigm’.^12^

In accordance with the Institute of Medicine naming patient‐centred care a key quality domain,^13^ quality of care for patients with severe AS may be improved through the elicitation and inclusion of patient‐defined goals in both delineation of choices and in treatment selection. We describe here a first step: the elicitation and reporting of patient‐defined goals for the treatment of severe AS in high‐risk patients.

## Methods

### Setting and participants

This retrospective analysis was conducted by members of the multidisciplinary ‘Heart Team’ of the Heart and Vascular Center at Dartmouth‐Hitchcock Medical Center, a tertiary academic medical institution in Northern New England. The Heart Team, defined broadly, may include interventional cardiologists, cardiac surgeons, valvular heart disease clinicians, multimodal imaging experts, palliative care physicians and nurses, among other administrative and clinical support staff.^14^ Patients included in this study were elderly and either high‐risk for SAVR or were inoperable. All patients had severe AS and were eligible for TAVR following initial Heart Team evaluation between June 2012 and August 2014.

### Data collection

The TAVR programme coordinator is a master's‐level nurse on the Heart Team, responsible for assisting in the assessment of patient eligibility for both research and clinical application of TAVR, and facilitating discussion with the patient and family regarding processes of care.^14^ The TAVR coordinator's primary goal is to assist patients who have a high likelihood of undergoing TAVR and their families; thus, the clinical practice described here does not encompass the entirety of patients undergoing care for severe AS at Dartmouth‐Hitchcock Medical Center, but represents a subset of high‐risk or inoperable patients co‐managed by a dedicated nurse coordinator.

**Figure 1:**
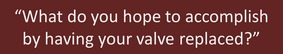
Question delivered in the clinical visit to elicit patient‐defined goals for TAVR. The TAVR coordinator asked successive patients a question designed to elicit each patient's uniquely defined goal for considering TAVR. TAVR, transcatheter aortic valve replacement.

In June 2012, a quality improvement initiative was begun by the TAVR coordinator through which she met with every patient undergoing consideration of TAVR and elicited patient goals by asking the following question during the initial evaluation: ‘What do you hope to accomplish by having your valve replaced?’ (Fig. 1) When family members answered for the patient, the question was redirected to the patient for further elaboration.

Patient‐defined goals were documented within an internally protected spreadsheet by the TAVR programme coordinator and were subsequently discussed at Heart Team conferences in the context of patient risk profiles. Information tracking the patients’ perceptions of having met their goals following procedure was recorded dichotomously as a ‘yes’ or ‘no’ response. For patients with missing data, detailed review of the medical record was conducted by the TAVR coordinator to assess and categorize goals for therapy. Informed consent was obtained from patients and family members at the time of analysis for the use of identifying patient materials (including video) (Video S1). The Dartmouth College Center for the Protection of Human Subjects approved this analysis.

### Data analysis

We conducted a retrospective, qualitative review of the patient‐defined goals elicited and documented from June 2012 through August 2014. Noting the range of themes conveyed by the patients in communicating their personal treatment goals, we formulated four descriptive groups in which to categorize the patient‐defined goals. These categories were adapted from prior work describing the analysis of patient prioritization of health outcomes in a similar, elderly patient population: (i) maintaining independence; (ii) staying alive; (iii) reducing/eliminating pain or symptoms; and (iv) ability to do a specific activity.^15^

The de‐identified patient‐defined goals were categorized by three independent reviewers (MC, EO and RP) and categorizations compared. Discrepancies were resolved through discussion among the reviewers.

Attainment of goals for TAVR was assessed by the study team through electronic medical record review. One‐month follow‐up visit notes were reviewed, along with the notes written by the TAVR coordinator at the time of the follow‐up visit.

Detailed patient characteristics are recorded as part of the internal TAVR programme as well as the Transcatheter Valvular Therapies (TVT) registry. The Society of Thoracic Surgeons (STS) operative risk score was calculated utilizing the online calculator (http://riskcalc.sts.org/). Kansas City Cardiomyopathy Questionnaire (KCCQ‐12) scores were measured at the initial visit and again 30 days following the procedure, utilizing the required standardized questionnaire for the TVT registry (https://www.ncdr.com/TVT/Home/DataCollection.aspx).

## Results

### Patient characteristics

Characteristics of the 46 participants are provided in Table 1. The majority (89%) of patients were 75 years of age or older, with a mean age of 84 years. One patient was 100 years old at the time of procedure. Over half of the patients were men (54%). The mean STS score recorded was 9%. Most patients who underwent TAVR were discharged to their homes (61%): independently, with family, or with a visiting nurse association service. One‐third of the patients (35%) underwent rehabilitation following their hospital stay. Two patients (4.3%) died in the hospital. The mean KCCQ‐12 score at baseline was 36 (range 4–76) and at 30 days following TAVR was 77 (range 22–100). Follow‐up for patients occurred 3–6 weeks after procedure.

**Table 1:** Characteristics of patients who underwent transcatheter aortic valve replacement and reported treatment goals (*n* = 46)

Characteristic	Number (%) or mean ± SD^a^
Sex	
Male	25 (54.3)
Female	21 (45.7)
Age	84 ± 7.2
68–74	5 (10.9)
75–89	29 (63.0)
90+	12 (26.1)
STS^b^ score	9 ± 4.9
3–9	29 (63.0)
10+	15 (32.6)
Not reported	2 (4.3)
KCCQ^c^ score, initial	36 ± 15.9
KCCQ^c^ score, 30 days	77 ± 18.8
Days to procedure	60 ± 41.8
Discharge status	
Home	24 (52.2)
Home with VNA^d^	4 (8.7)
Rehabilitation	2 (4.3)
Rehabilitation to home	13 (28.3)
Rehabilitation to assisted living	1 (2.2)
Deceased before discharge	2 (4.3)
Year procedure performed	
2012	3 (6.5)
2013	14 (30.4)
2014 (through August)	29 (63.0)

a Standard deviation.

b Society of Thoracic Surgeons.

c Kansas City Cardiomyopathy Questionnaire.

d Visiting nurse association.

Seven patients eligible for TAVR eventually chose not to undergo the procedure and selected medical management/palliative care. Patient characteristics are provided in Table 2. The mean age was 86 years, and over half were female. During a follow‐up period of one year, three patients died.

**Table 2:** Characteristics of patients eligible for transcatheter aortic valve replacement who chose medical therapy/palliative care (*n* = 7)

Characteristic	Number (%) or mean ± SD^a^
Sex	
Male	2 (25.0)
Female	5 (62.5)
Age, years	86 ± 4.5
75–89	6 (75.0)
90+	1 (12.5)

a Standard deviation.

### Patient‐defined goals

#### Maintaining independence

**Figure 2:**
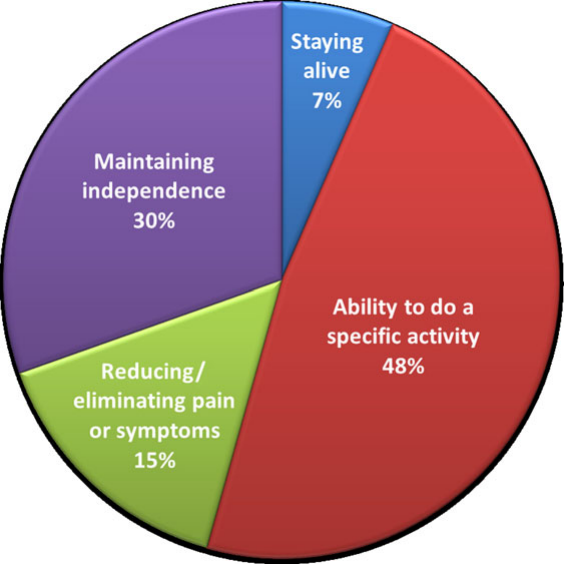
Categories of patient‐defined goals for TAVR. Patient‐defined goals for TAVR were categorized based on elderly patients’ prioritizations of health outcomes. TAVR, transcatheter aortic valve replacement.

For just under one‐third of patients, (14 patients, 30%) maintaining or improving their current level of independence was the desired outcome of interest when considering TAVR (Fig. 2). Many patients were previously living alone and due to their valvular heart disease, were no longer able to maintain their homes or do daily chores such as cleaning, gardening, chopping wood or grocery shopping. As one patient explained:

I want to be able to garden, walk, do household chores… I want to live in my home independently.

Most patients wanted to ‘remain in an independent living setting’ or simply ‘stay in [their] home[s]’.

#### Staying alive

A small minority of patients (three patients, 7%) stated that their primary goal in pursuing treatment was to simply remain alive (Fig. 2). There was an acknowledgement of the mortality associated with medical therapy in the context of the natural history of AS, and this feature weighed most heavily on their communicated goals. Patients expressed this primary outcome often in clear, concise statements:

I want to live.

We observed that increasing the length of life for patients was often a preference expressed by the adult children, and not by the patients themselves. Goals focused around survival initially stated by family members were redirected to the patient for further clarification.

#### Reducing/eliminating pain or symptoms

Seven patients (15%) reported that reduction of pain, symptoms and suffering was their primary goal for considering valve replacement (Fig. 2). Of these seven patients, three patients desired treatment for AS given that it was a prerequisite to undergo additional procedures to relieve suffering, including orthopaedic surgery and tumour resection.

For those patients that expressed desire of relief of suffering from the symptoms of AS, the most consistent complaint was of profound, persistent fatigue:

I want to have more ambition. Right now I don't have the energy to do anything. I sleep all the time.

Another patient expressed how restricting the limitations were as a result of feeling unwell:

I want to feel better. I can't do anything right now.

#### Ability to do a specific activity

The most frequently reported goal from patients was that of the ability to do a specific activity: nearly half (48%) of patients communicated this finding (Fig. 2). Many expressed wanting to participate in the daily hobbies they could no longer enjoy because of their illness. Most of the activities centred on how patients interacted with their loved ones, as one patient described:

I want to be able to take my wife out, ride my motorcycle, go fishing…

The desire to regain the stamina to enjoy personal activities that defined the patient's persona was also prominent:

I love to dance; I just want to dance a few more dances.

Patients wanted to resume volunteer work and enjoy time spent with their spouses, grandchildren and favourite pets. Many patients were fond of hiking, biking or walking. Others enjoyed doing arts and crafts. One patient wanted to write a book. There were several patients that were interested in travel:

I want to travel to Italy.

Together, these patient‐defined goals highlighted specific activities with others, as well as personal hobbies, that were central to consideration of valve replacement for each patient.

#### Patients who decided against TAVR

Seven of the patients eligible for TAVR during the specified time period chose not to undergo the procedure after initial consideration, and instead chose to continue medical therapy with additional input from palliative care. One of these patients had early Parkinson's disease:

I would rather die quickly of heart failure… Knowing what my options are with Parkinson's, I don't want to face a prolonged, debilitated life.

The remaining patients reported the goal of avoiding invasive procedures and potential complications. One patient expressed a concern of the possible need for blood transfusion during the procedure; this patient was an active Jehovah's Witness. Others feared stroke, possibility of dialysis and prolonged need for ventilation.

Also present was a focus on maintaining control around end‐of‐life planning:

I want to go home and get my affairs in order. I don't want anything else.

### Follow‐up

The majority of patients who underwent TAVR (87%) had achieved their stated goals at one month, determined by descriptive visit notes and documented New York Heart Association functional status. For example, two patients with goals of being more active quantified goal achievement with specific accomplishments: one recognized she was walking nearly four times the distance at one month than she was before her TAVR, and another acknowledged a newfound ability to exercise.

We concluded that six (13%) patients’ goals were not met. Two patients died in the hospital before being discharged, and two patients had severe symptoms at one‐month follow‐up and passed away within four months of being discharged. One patient continued to feel debilitated and fatigued, limiting his ability to participate in his favourite activities; his dyspnoea hindered him from achieving his goal. Similarly, another patient whose goal was unattained hoped to enjoy outdoor hobbies; the patient continued to feel increasingly fatigued at one‐month follow‐up.

## Discussion

We found that eliciting patient‐defined goals among elderly patients considering TAVR is feasible with a simple question delivered in the clinical setting. Notably, many patients communicate their preferred treatment goals based upon their ability to do a specific activity, rather than around specific symptomatology. Current measures of quality of life for patients with AS, such as the KCCQ‐12, are focused on evaluation of the effectiveness of the selected therapy in the context of symptoms such as shortness of breath, chest pain and syncope, symptoms not consistently mentioned by our patients in goal setting; quantitative measures like these may not be capturing what is most important to patients.

The existing literature shows that most patients (96%) want their available treatment options presented to them, in addition to being asked their opinions regarding those options.^16^ However, patients often feel either uncomfortable or not empowered to express their goals regarding potential treatment options during clinical visits.^17^ Prior work in goal setting in other clinical contexts has shown that there are often discrepancies among patient‐ and clinician‐defined goals of care and that shared decision making could provide a framework to align goals and improve outcomes.^18^ We found that patients can (and are willing to) define personal goals for their care, if they are asked to do so.

This is consistent with prior research, showing that elderly patients are capable not only of expressing what is important to them, but also of prioritizing the relative importance of various health outcomes.^15^ Maintaining independence is shown to be consistently important to elderly patients faced with multiple treatment options.^19^, ^20^, ^21^ Patients were found to most often define goals around specific activities; this finding is similar to results of prior work.^22^

Notably, research on decision making in older adults has been inconclusive regarding the most effective methods for facilitating the elicitation of patient preferences. Successful decision making requires a discussion between the clinician and the patient about comparable treatment options framed by the patient's preferences and values regarding those options.^5^ Elicitation of patients’ preferences about their personal goals for treatment, not just about the salient risks and benefits, is an essential element of shared decision making; successful elicitation of goals for therapy, together with prioritization of what matters most to patients, may contribute to more accurate ‘preference construction’.^23^ We found that the elicitation of patient‐defined goals was obtainable with a straightforward, open‐ended question delivered by a member of the Heart Team. By asking patients about their treatment goals, clinicians can expand their ability to effectively elicit patient preferences. Improving physician competence in this area is a critical step in beginning to engage clinicians in successful implementation of shared decision making.^24^

### Implications for further research

Training of physicians on how to elicit patient values and preferences with the limited time available in the clinical visit through simple questions, such as that utilized here, may help to incorporate patient values and goals into conversations about comparable treatment options and their associated risks and benefits. Recognizing how elderly patients understand questions regarding preferred treatment goals, and their prioritization of those goals, informs the decision making process and can aid the development of strategies to support clinician engagement in shared decision making.

### Study limitations

By nature of our retrospective analysis, this study may be subject to selection bias, as the study population had already expressed treatment goals at the time of study inclusion and data analysis. Given the lack of prospective data collection, audio recording was not performed. The TAVR coordinator recorded patient‐defined goals utilizing an internal spreadsheet; in the context of the retrospective nature of the study, there is a potential for both patient selection and recall bias. A larger sample size of both clinicians and patients is needed to confirm the results of this analysis.

## Conclusions

Elderly patients with severe AS are able to define personal and unique goals when considering treatment options. We found that the most common patient‐defined goal is the ability to enjoy favourite activities and spend time with loved ones rather than goals related to heart failure symptomatology. Patient‐centred goals for therapy may inform selection of treatment options aligned with patient preferences; elicitation of these preferences is paramount to the continued movement towards increased patient‐centred care. Diverse members of the Heart Team, including nurses, can be successful in eliciting patient preferences.

## Source of funding

None.

## Conflict of interests

No conflicts of interest have been declared.

## Supporting information


**Video S1**. Elicitation of a patient's goal for treatment of severe aortic stenosis using a decision aid.
